# Exploring the potential of Oxford Nanopore Technologies sequencing for *Mycobacterium tuberculosis* sequencing: An assessment of R10 flowcells and V14 chemistry

**DOI:** 10.1371/journal.pone.0303938

**Published:** 2024-06-06

**Authors:** Anzaan Dippenaar, Emilyn Costa Conceição, Felicia Wells, Johannes Loubser, Brendon Mann, Miguel De Diego Fuertes, Vincent Rennie, Robin Mark Warren, Annelies Van Rie

**Affiliations:** 1 Department of Family Medicine and Population Health, Global Health Institute, Faculty of Medicine and Health Sciences, University of Antwerp, Antwerp, Belgium; 2 Division of Molecular Biology and Human Genetics, South African Medical Research Council Centre for Tuberculosis Research, Faculty of Medicine and Health Sciences, Stellenbosch University, Cape Town, South Africa; Centre de Recherche en Cancerologie de Lyon, FRANCE

## Abstract

Oxford Nanopore Technologies (ONT) sequencing is a promising technology. We assessed the performance of the new ONT R10 flowcells and V14 rapid sequencing chemistry for *Mtb* whole genome sequencing of *Mycobacterium tuberculosis* (*Mtb*) DNA extracted from clinical primary liquid cultures (CPLCs). Using the recommended protocols for MinION Mk1C, R10.4.1 MinION flowcells, and the ONT Rapid Sequencing Kit V14 on six CPLC samples, we obtained a pooled library yield of 10.9 ng/μl, generated 1.94 Gb of sequenced bases and 214k reads after 48h in a first sequencing run. Only half (49%) of all generated reads met the Phred Quality score threshold (>8). To assess if the low data output and sequence quality were due to impurities present in DNA extracted directly from CPLCs, we added a pre-library preparation bead-clean-up step and included purified DNA obtained from an *Mtb* subculture as a control sample in a second sequencing run. The library yield for DNA extracted from four CPLCs and one *Mtb* subculture (control) was similar (10.0 ng/μl), 2.38 Gb of bases and 822k reads were produced. The quality was slightly better with 66% of the produced reads having a Phred Quality >8. A third run of DNA from six CPLCs with bead clean-up pre-processing produced a low library yield (±1 Gb of bases, 166k reads) of low quality (51% of reads with a Phred Quality score >8). A median depth of coverage above 10× was only achieved for five of 17 (29%) sequenced libraries. Compared to Illumina WGS of the same samples, accurate lineage predictions and full drug resistance profiles from the generated ONT data could not be determined by TBProfiler. Further optimization of the V14 ONT rapid sequencing chemistry and library preparation protocol is needed for clinical *Mtb* WGS applications.

## Introduction

Tuberculosis (TB), caused by *Mycobacterium tuberculosis* (*Mtb*), continues to pose a significant global health challenge. Antibiotic resistance worsens treatment outcomes, underscoring the urgency of the implementation of accurate, efficient, and comprehensive diagnostic strategies such as Next-Generation Sequencing (NGS) [1]. Long read sequencing approaches from Oxford Nanopore Technologies (ONT) have been successfully used for *Mtb* whole genome sequencing (WGS) [2–5], and emerged as a promising approach due to its small size and relatively low capital costs allowing for decentralized use and rapid processing times which leads to real-time sequencing capabilities. The use of a targeted ONT sequencing assay (NanoTB®) was recently endorsed by the World Health Organization (WHO) for the detection of drug resistance based on promising results in the Seq&Treat study [6]. However, NanoTB®, due to its targeted methodology, does not detect all candidate drug resistance variants listed in the second edition of the WHO catalogue of drug-resistance mutations in *Mtb*, and does not allow *Mtb* transmission inference [7].

While ONT WGS of *Mtb* has been successful in research studies, its use on clinical specimens or clinical primary liquid cultures (CPLCs) is currently hindered by the need for high DNA input amounts (400–1000 ng per sample) when using the SQK-LSK109 ONT kit [8–10]. In contrast to the yield from *Mtb* subcultures used in research studies, such high DNA input amounts are rarely achieved when extracting DNA from CPLCs [10, 11]. Using CPLCs would be beneficial for clinical *Mtb* WGS as it has a faster turnaround time compared to a subculture and could better preserve *Mtb* population diversity [12, 13].

For samples with low DNA yield, if opting for the ONT ligation sequencing approach, ONT recommends fragmentation of 100 ng of DNA by either sonication or a mechanical method [14], which requires costly equipment (e.g. FastPrep, Megaruptor 3) [15, 16] or spin-column-based fragmentation such as the COVARIS G-tube methodology [17], which is expensive (e.g a single COVARIS G-tube costs >50 EUR and can only be used for a single sample). The ONT ligation sequencing library preparation protocol is also labour-intensive, especially when multiplexing samples, which requires additional steps for the inclusion of native barcoding. A multiplexed ligation sequencing preparation protocol requires up to eight hours of hands-on time from a skilled laboratory technician to prepare 4–12 samples, limiting scalability. While automation alternatives, such as ONT VolTRAX may mitigate this challenge [18], VolTRAX automation is not yet fully developed for all ONT protocols [19, 20]. Furthermore, ONT flowcell versions, such as R9, had reduced per-base sequence accuracy (>95%) in comparison to highly precise short-read Illumina platforms that achieve accuracy rates exceeding 99% [21, 22]. The low accuracy has limited the use of ONT WGS for outbreak analysis, as identification of all genetic variants present in *Mtb* genomes is paramount for transmission inference and clinical drug resistance detection and single base errors could also influence drug resistance predictions [9]. Finally, the ligation sequencing protocols require costly third-party reagents, increasing per-sample cost, and the multiple bead clean-up steps integrated into the protocol can reduce the DNA yield in the final sequencing library, which further exacerbates the need for high DNA input [23].

To address some of these limitations, ONT developed R10 flowcells, which have per-base sequence accuracy similar to that of Illumina sequencing, and introduced the Rapid Sequencing Kit V14 [24]. This kit requires less DNA input (50 ng per sample), allows for sequencing of as few as five multiplexed samples in a single run, does not require prior DNA fragmentation, and relies solely on the ONT reagents provided in the kit. It is also less time-intensive (approximately 60 minutes for the preparation of a multiplexed sequencing run). As these improvements could allow the adoption of ONT sequencing in routine clinical care, we sought to evaluate the performance of ONT R10 flowcells and V14 rapid sequencing chemistry for *Mtb* WGS of CPLCs.

## Methods

### Ethics statement

The samples (DNA extracted from clinical *Mycobacterium tuberculosis* isolates) originated from the SMARTT trial, which received ethical approval from the ethics committees of the University of the Free State Health Sciences Research Ethics Committee (UFS-HSD2019/0364/2004), Stellenbosch University Health Research Ethics Committee (N19/07/100), and the University of Antwerp (21/18/239 –also acted as study sponsor).

### Experimental overview

**Fig 1:**
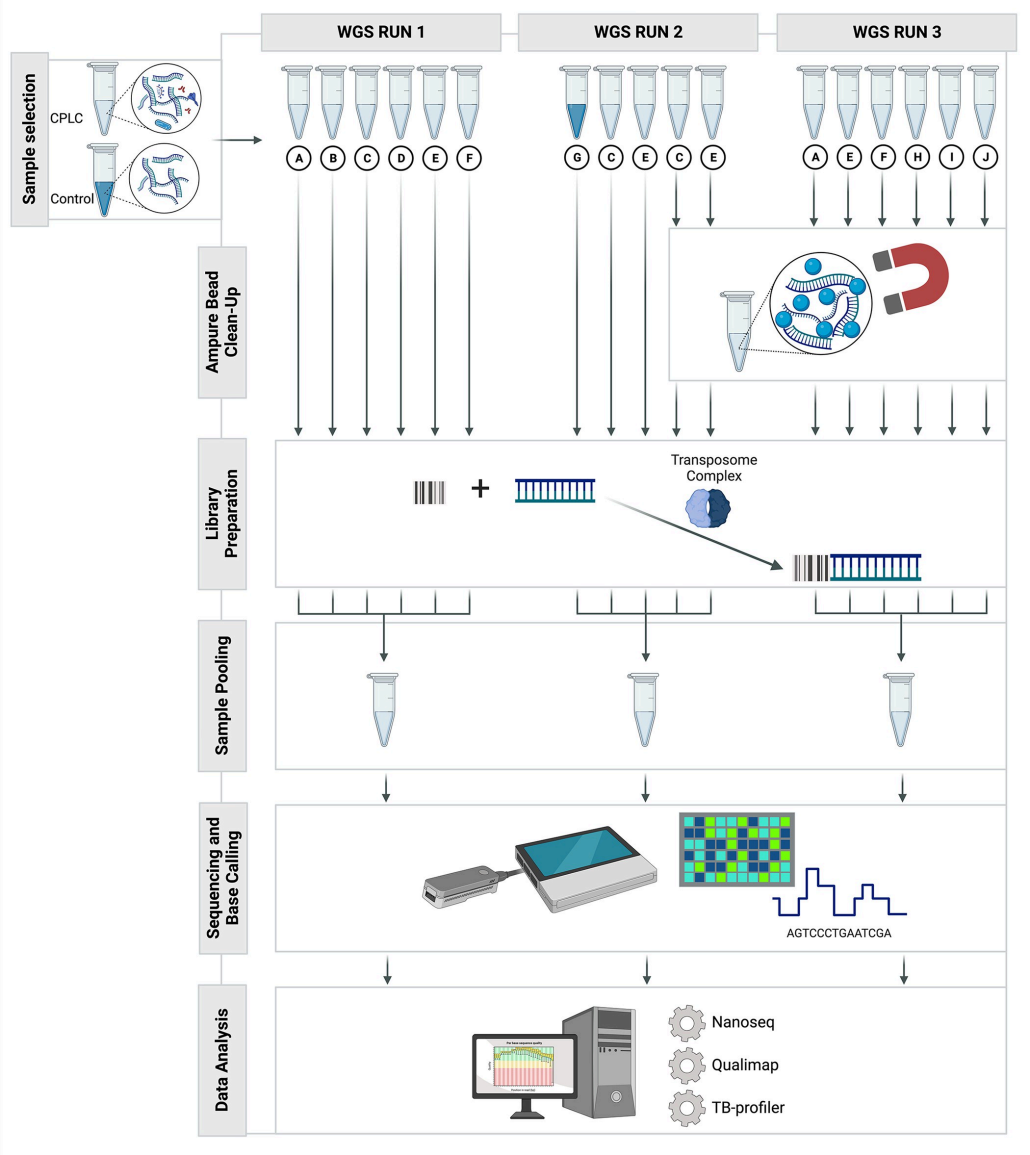
Experimental workflow for ONT sequencing of gDNA extracted from *Mtb* CPLC samples. A schematic representation of the experimental workflow, detailing the three sequencing runs, the number of samples multiplexed in each run, and the incorporation of a bead clean-up step for selected samples. The iterative approach involved data analysis after each run to guide adjustments and enhancements in subsequent runs, enabling the optimisation of the ONT sequencing process for *Mtb* CPLCs. Figure created with Biorender.com.

We assessed the quality of *Mtb* WGS of CPLCs using the ONT Rapid Sequencing Kit V14 for library preparation and sequencing on a MinION device connected to an MK1C instrument and used an iterative approach to optimise the experimental procedure of subsequent runs, using the obtained results to adjust the protocol for the following run (Fig 1).

### Sample selection and DNA extraction

We selected *Mtb* DNA extracted by a modified (reduced reagent volume and resuspension of DNA in 25 μl nuclease free water) cetyltrimethylammonium bromide (CTAB) method [25] of nine CPLCs from mycobacteria growth indicator tubes (MGITs) from patients diagnosed with rifampicin-resistant TB who participated in the SMARTT clinical trial [26]. On average, the tubes flagged positive for mycobacterial growth by the MGIT 960 instrument at 13.6 days after inoculation (ranging from 5 to 36 days). After flagging positive, cultures were incubated at 37°C for an additional 7.1 days on average (ranging from 1 to 20 days). For the research presented in this paper, the data were accessed in February 2023, no clinical data or personal information were used in this analysis. Samples were eligible for inclusion in this study when more than 60 ng of the originally extracted DNA were available, and Illumina WGS data derived from the same sample had passed quality control as part of the MAGMA bioinformatics analysis [27]. Based on Illumina WGS analysis, these samples contained varying levels of non-*Mtb* contamination (73% median percentage of reads mapped to the *Mtb* reference genome, H37Rv) and actionable Illumina WGS results (drug resistance prediction) with a median genome-wide depth of coverage ≥52× (median 106, S1 Table).

We introduced a control *Mtb* DNA sample (G) extracted from a sub-cultured clinical *Mtb* isolate in WGS Run 2. This control sample had previously undergone Illumina WGS as well as ONT WGS using the Ligation sequencing kit with native barcoding.

### Library preparation and sequencing

We adhered to the manufacturer’s protocol for library preparation using the ONT Rapid Sequencing Kit V14 (SQK-RBK114). Samples A-F were used for WGS Run 1 without prior bead clean-up of the DNA (Fig 1). In WGS Run 2 and WGS Run 3, we introduced a pre-library preparation bead-clean-up step using Agilent Ampure XP beads to remove potential impurities in DNA extracted from CPLCs. WGS Run 2 comprised the control sample (G) and two CPLC DNA samples (C and E), with one aliquot included in the library preparation without bead clean-up, and another aliquot after undergoing bead clean-up. For all samples involved in WGS Run 3 (A, E, F, H, I, J), bead clean-up was performed prior to library preparation and sequencing. Briefly, the DNA sample was mixed with 1× volume of Ampure XP beads and incubated for 5 minutes to allow the DNA to bind to the beads. After capturing the DNA-bound beads using a magnet, the beads were washed twice with 80% ethanol, as per the manufacturer’s instructions. DNA was eluted from the beads using 11 μl nuclease-free water.

All sequencing runs were performed using R10.4.1 MinION flowcells (FLO-MIN114) on an ONT MinION MK1C (MIN-101C) sequencing platform, according to the manufacturer’s protocol.

### Data analysis

Raw ONT data (FAST5) were base-called using MinKNOW software (v21.05.12) on super high accuracy, filtered on Phred Quality score 8, and saved in the FASTQ format. Base-calling also removed barcodes and adaptors. Generated data were analysed using nfcore/nanoseq (v3.1.0) [28, 29], specifying the *Mtb* H37Rv (NC_000962.3) sequence as a reference, and using Deepvariant (v1.4.0) [30] for variant identification. Qualimap 2 (v2.2.2-dev) was used to estimate the mapping statistics of the mapping files outputted by the nfcore/nanoseq (v3.1.0) analysis [31, 32]. The variant files produced by Deepvariant (v1.4.0) were further processed using TBProfiler (v6.2.0) to produce annotations of drug-resistance conferring variants and lineage identification, specifying ‘nanopore’ as the platform [33, 34]. TBProfiler was run using default parameters, with the exception of the “platform” parameter, which was set to ‘nanopore’ to indicate the sequencing platform used for data generation. Kraken 2 was used for taxonomic classification of the raw data [35].

## Results

### Performance metrics of multiplexed ONT sequencing runs for *Mtb* WGS

For WGS Run 1, we obtained a pooled library concentration of 10.9 ng/μl. During the 48-hours sequencing run, 1.94 gigabases (Gb) of data and 214,000 reads were produced. Nearly half of the reads (49%) had low sequencing quality as they did not meet the minimum Phred Quality score threshold of >8 (Table 1).

**Table 1:** Performance metrics of multiplexed ONT sequencing runs for *Mtb* WGS.

	RUN 1	RUN 2	RUN 3
Number of samples pooled	6	5	6
Pooled library concentration	10.9 ng/μl	10.0 ng/μl	13.7 ng/μl
Amount of DNA library loaded on flow cell	130.8 ng	120 ng	164.4 ng
Flowcell check active pores	±1800	±1600	±1300
Estimated bases generated	1.94 Gb	2.38 Gb	997.71 Mb
Reads generated	213.84 k	822 k	165.54k
% of Reads passing QC (Phred 8)	49%	66%	51%

Despite the introduction of an extra bead clean-up step for two of the four CPLC DNA samples to overcome potential impurities in the CPLCs, the library concentration of WGS Run 2 (10.0 ng/ μl) was comparable to that obtained in WGS Run 1. WGS Run 2 produced 2.38 Gb of data and 822,000 reads, a higher data yield compared to WGS Run 1. There was only a small improvement in the overall sequencing quality, with 66% of reads having a Phred Quality score >8.

In WGS Run 3, where all samples were pre-processed using a bead clean-up step, the output was lower, generating only ±1 Gb of data and 166,000 reads, suggesting low flow cell efficiency. The read quality did not improve compared to WGS Run 2 with 51% of the reads having a Phred Quality score >8. WGS Run 3 showed signs of poorer flow cell performance.

### Per sample WGS quality control metrics

The 17 sequenced samples displayed variations in the depth of genome coverage. Only five samples had a median depth of coverage >10× (Table 2). In WGS Run 1, three out of six samples achieved the 1× coverage breadth threshold (at least one read is present for 90% of the reference *Mtb* genome), and one sample achieved the 5× coverage breadth threshold. In WGS Run 2, all five samples achieved the 1× coverage breadth threshold, and four of the five samples the 5× coverage breadth threshold. The effect of bead clean-up was noticeable in WGS Run 2, as only one sample did not meet the 5× coverage breadth threshold (C without bead clean-up), while the same sample that did undergo bead clean-up in this run (C-BCU) and all other samples in the run met the threshold. In WGS Run 3, one sample met the 1× threshold but none of the samples achieved the 5× coverage breadth threshold.

**Table 2:** Per-sample ONT WGS metrics for *Mtb* analysis.

Sample ID	ONT WGS run	Bead clean-up	Percentage mapped reads (%)	Number of mapped reads	Median depth of coverage (×)	Breadth of coverage: ≥1× depth of coverage (%)	Breadth of coverage: ≥5× depth of coverage (%)
A	1	no	88.65	3475	2	87.7	8.7
B		no	58.18	921	0	45.8	0
C		no	87.51	25620	11	99.6	95.9
D		no	85.57	26761	4	96.2	27.1
E		no	90.85	9825	3	90.2	10.2
F		no	86.29	2764	0	48.6	0.1
G-control	2	NA*	99.1	96947	29	99.3	99.2
C-BCU		yes	90.21	80064	29	99.7	99.6
C		no	89.47	17518	5	98.8	53.8
E-BCU		yes	94.58	80586	20	99.5	99.2
E		no	93.84	85691	20	99.4	99.2
A-BCU	3	yes	89.71	2292	1	70.8	1.1
H-BCU		yes	97.49	5253	1	64.5	0.3
E-BCU		yes	85.1	23053	5	98.2	50.8
F-BCU		yes	83.29	13513	2	89.8	10.3
I-BCU		yes	36.52	5988	1	63.1	0.5
J-BCU		yes	87.46	3900	2	81.9	3.3

*The DNA used for sample G was extracted from a subcultured *Mtb* clinical isolate using the modified CTAB method and was added as a control to sequencing run 2. It’s important to note that this particular sample, distinct from CPLCs, has previously been successfully subjected to both Illumina and ONT sequencing using the ligation sequencing kit.

### Drug resistance prediction and strain identification

The ONT WGS analysis by TBProfiler assigned a lineage to all 17 samples that were sequenced (Table 3). However, in WGS Run 1, the lineage identification using ONT WGS data differed from the Illumina WGS data, with concordance for only one sample (C). The ONT WGS lineage detection for all samples in WGS Run 2 agreed with the Illumina-based lineage prediction. In WGS Run 3, one sample (F) was identified as a potential mixed infection by ONT while the Illumina WGS identified a single lineage (lineage 2.2.1), only one sample in this run matched the Illumina-based lineage prediction. For drug resistance prediction, ONT consistently underreported the level of drug resistance. In all samples but one, Kraken 2 classified the majority of reads (>50%) as belonging to the *Mycobacterium tuberculosis* complex (MTBC), with varying proportions categorised as unclassified or other. Detailed results are provided in S2 Table.

**Table 3:** Comparison of ONT- and Illumina-based lineage prediction and drug resistance profiling.

Sample	Run	ONT		Illumina	
		Lineage	Drug resistance	Lineage	Drug resistance
A	1	4.9	Susceptible	2.2.1	RR-TB
B		4.9	Susceptible	4.4.1.1	RR-TB
C		4.4.1.1	Susceptible	4.4.1.1	Pre-XDR-TB
D		4.9	Susceptible	4.3.2.1	XDR-TB
E		4.9	Susceptible	4.1.1.3	MDR-TB
F		4.9	Susceptible	2.2.1	XDR-TB
G-control	2	2.2.2	Pre-XDR-TB	2.2.2	Pre-XDR-TB
C		4.4.1.1	Other	4.4.1.1	Pre-XDR-TB
C-BCU		4.4.1.1	MDR-TB	4.4.1.1	Pre-XDR-TB
E		4.1.1.3	RR-TB	4.1.1.3	MDR-TB
E-BCU		4.1.1.3	MDR-TB	4.1.1.3	MDR-TB
A-BCU	3	4.9	Susceptible	2.2.1	RR-TB
H-BCU		4.9	Susceptible	4.1.1.3	MDR-TB
E-BCU		4.1.1.3	Susceptible	4.1.1.3	MDR-TB
F-BCU		4.9; 2	Susceptible	2.2.1	XDR-TB
I-BCU		4.9	Susceptible	2.2.1	MDR-TB
J-BCU		4.9	Susceptible	4.3.2.1	RR-TB

BCU = bead clean-up

## Discussion

In this study, we encountered several hurdles to performing ONT WGS from CPLC DNA samples and observed suboptimal sequencing quality, low data output, and inadequate genome coverage for accurate lineage identification and drug resistance prediction. These challenges occurred despite efforts to optimise library preparation and sequencing protocols and appeared to be intricately linked to the need for high concentrations of pure DNA, which is hard to achieve when using DNA extracted from CPLCs. Achieving sufficient depth of coverage is crucial for generating reliable and actionable *Mtb* WGS results [27, 36]. Having a median depth of coverage <10× introduces uncertainty and raises the risk of misidentifying genetic variants, which is detrimental in the context of clinical drug resistance detection as shown by the incorrect classification of 70,5% (12/17) of the included *Mtb* strains as pan-susceptible. Analysis of the ONT WGS data also struggled to correctly identify the *Mtb* lineage, with the identified lineage 4.9 (the same as the *Mtb* reference genome, H37Rv) in nine samples identified as another lineage by analysis of the Illumina sequencing data. This is most likely due to the less accurate detection of lineage markers in samples with insufficient depth and breadth of coverage and the interpretation of the absence of variants as being the same as the reference genome, H37Rv, belonging to lineage 4.9. The findings of this study underscore the challenges inherent to the adoption of current ONT sequencing for *Mtb* whole genome analysis in a clinical context.

Additionally, we used primary MGIT cultures obtained from inoculation directly from the decontaminated sputum specimens, thereby bypassing a subculture step, for DNA extraction. Clinical samples often introduce a degree of inconsistency due to contamination, impurities, and disparities in DNA quality across samples. This inherent variability within clinical samples can influence the reliability and accuracy of the sequencing data, making interpretation of results challenging, especially in studies with small sample sizes. While bead clean-up to purify DNA can overcome some of these issues like removing small DNA fragments, salts and other contaminants or impurities, it did not result in a substantial gain in sequence quality or data output. Striking a balance between thorough sample cleaning and retaining the maximal DNA quantity will be critical for sequencing CPLCs using ONT sequencing. Despite using a bead clean-up for all samples, the third sequencing run revealed an unexpectedly low data output. This was likely due to technical issues with an underperforming flow cell, or other variables beyond DNA quality. Further work should explore whether only loading samples that underwent bead clean-up or a similar DNA purification step can generate data of sufficiently high quality for clinical *Mtb* WGS when using V14 ONT rapid sequencing chemistry.

Communication with ONT’s technical support team brought attention to a potential cause for the low output and quality of ONT WGS data, suggesting that underloading of flow cells might have occurred. This occurred despite our strict adherence to the published official protocol, aligning with concerns raised by others on the ONT community page regarding the new V14 rapid barcoding chemistry. The manufacturer recommends multiplexing of four barcodes at minimum, we consistently used at least five. The protocol specifies that a maximum of 800 ng of a high-quality library can be loaded onto a MinION flow cell [24], we loaded 130.8 ng, 120 ng and 164.4 ng, respectively in the three WGS runs performed. The Rapid Sequencing Kit V14 protocol does not specify a minimum amount of library to be loaded. Future studies of clinical *Mtb* WGS using ONT could assess if multiplexing larger numbers of samples leads to higher library concentrations and potentially better data output.

Our results suggest that the ONT rapid sequencing technology may not yet be robust enough for clinical *Mtb* WGS. Negative findings or the challenges faced in implementing sequencing technologies play a significant role in promoting transparency but often receive less attention in the scientific literature due to publication bias. Publication of challenges can highlight the need for further improvement of existing technologies.

## Conclusion

We found that the quality of the sequencing data produced by ONT R10 flowcells with the new V14 rapid sequencing chemistry for WGS of *Mtb* DNA extracted from CPLC samples was mostly insufficient for accurate lineage and drug resistance identification. Our results emphasise the need for ongoing optimisation efforts in ONT sequencing strategies for WGS from clinical *Mtb* samples. Addressing the challenges could facilitate the successful implementation of ONT WGS in clinical settings, ultimately contributing to more effective TB care and prevention.

## Supporting information

S1 Table ONT WGS run information. (DOCX)

S2 Table Kraken 2 analysis. (DOCX)
