# Supplementary material for: Exploring the potential of Oxford Nanopore Technologies sequencing for Mycobacterium tuberculosis sequencing: An assessment of R10 flowcells and V14 chemistry
Source: PLoS One. 2024 Jun 6;19(6):e0303938. doi: 10.1371/journal.pone.0303938 (PMC11156342; doi:10.1371/journal.pone.0303938)
Supplement: S1 Table — (DOCX) [file pone.0303938.s001.docx]

| **Table S1** | | | | | |
| --- | --- | --- | --- | --- | --- |
| **Manuscript sample ID** | **WGS run1 barcode** | **WGS run2 barcode** | **WGS run3 barcode** | **Illumina Mapping %** | **Illumina Median Coverage** |
| A | RB01 |  | RB12 | 55.42 | 100 |
| B | RB02 |  |  | 61.24 | 112 |
| C | RB03 | RB08, RB09 |  | 70.94 | 92 |
| D | RB04 |  |  | 80.52 | 309 |
| E | RB05 | RB10, RB11 | RB14 | 78.1 | 140 |
| F | RB06 |  | RB15 | 67.83 | 216 |
| G |  | RB07 |  | 98.31 | 97 |
| H |  |  | RB13 | 75.24 | 124 |
| I |  |  | RB16 | 38.79 | 52 |
| J |  |  | RB17 | 79.98 | 76 |
|  |  |  |  |  |  |
|  |  |  | *min* | *38.79* | *52* |
|  |  |  | *max* | *98.31* | *309* |
|  |  |  | *ave* | *70.637* | *131.8* |
|  |  |  | *med* | *73.09* | *106* |
|  |  |  | *q1* | *62.8875* | *93.25* |
|  |  |  | *q3* | *79.51* | *136* |
|  |  |  | *IQR* | *16.6225* | *42.75* |
