# Supplementary material for: Exploring the potential of Oxford Nanopore Technologies sequencing for Mycobacterium tuberculosis sequencing: An assessment of R10 flowcells and V14 chemistry
Source: PLoS One. 2024 Jun 6;19(6):e0303938. doi: 10.1371/journal.pone.0303938 (PMC11156342; doi:10.1371/journal.pone.0303938)
Supplement: S2 Table — (DOCX) [file pone.0303938.s002.docx]

| **Table S2** | | | | | | |
| --- | --- | --- | --- | --- | --- | --- |
|  |  |  |  | **Kraken 2 raw read classification** | | |
|  | **WGS run #** | **Manuscript sample ID** | **ONT barcode** | **Unclassified** | **MTBC** | **Other** |
| **ONT** | WGS run 1 | A | RB01 | 4.97 | 85.33 | 9.7 |
|  |  | B | RB02 | 10.04 | 55.65 | 34.31 |
|  |  | C | RB03 | 14.26 | 80.08 | 5.66 |
|  |  | D | RB04 | 19.66 | 72.17 | 8.17 |
|  |  | E | RB05 | 8.84 | 83.68 | 7.48 |
|  |  | F | RB06 | 9.21 | 76.62 | 14.17 |
|  | WGS run 2 | G | RB07 | 2.02 | 97.08 | 0.9 |
|  |  | C | RB08 | 9.63 | 85.86 | 4.51 |
|  |  | C-BCU | RB09 | 8.34 | 87.35 | 4.31 |
|  |  | E | RB10 | 3.52 | 91.54 | 4.94 |
|  |  | E-BCU | RB11 | 3.11 | 92.52 | 4.37 |
|  | WGS run 3 | A-BCU | RB12 | 1.29 | 89.35 | 9.36 |
|  |  | H-BCU | RB13 | 1.28 | 96.55 | 2.17 |
|  |  | E-BCU | RB14 | 3.24 | 84.19 | 12.57 |
|  |  | F-BCU | RB15 | 4.57 | 81.67 | 13.76 |
|  |  | I-BCU | RB16 | 10.39 | 35.49 | 54.12 |
|  |  | J-BCU | RB17 | 1.91 | 86.75 | 11.34 |
|  |  |  |  |  |  |  |
|  | MTBC | *Mycobacterium tuberculosis* complex | |  |  |  |
|  | BCU | bead clean-up |  |  |  |  |
